# Study of the Combustion Mechanism of Zn/KMnO_4_ Pyrotechnic Composition

**DOI:** 10.3390/molecules28155741

**Published:** 2023-07-29

**Authors:** Mateusz Polis, Konrad Szydło, Roman Zakusylo, Lukasz Hawelek, Agnieszka Stolarczyk, Tomasz Jarosz

**Affiliations:** 1 Explosive Techniques Research Group, Łukasiewicz Research Network—Institute of Industrial Organic Chemistry, 42-693 Krupski Młyn, Poland; 2 Department of Physical Chemistry and Technology of Polymers, Silesian University of Technology, 44-100 Gliwice, Poland; 3 Shostka Institute, Sumy State University, 41100 Shostka, Ukraine; 4 Lukasiewicz Research Network— Institute of Non-Ferrous Metals, 5 Sowinskiego St., 44-100 Gliwice, Poland

**Keywords:** lead-free PDC, delay composition, pyrotechnic, detonators, combustion mechanism

## Abstract

This work aims to investigate the combustion mechanism for a pyrotechnic delay composition (PDC), consisting of zinc powder as a fuel and KMnO_4_ as an oxidising agent. For this purpose, the compositions were thermally conditioned at several set temperatures, chosen based on our previous work. Tests were also performed for post-combustion residues obtained via combustion of the PDCs in a manometric bomb. The samples were examined by scanning electron microscopy (SEM), Raman spectroscopy and X-ray diffractometry (XRD). Furthermore, the obtained results were correlated with previous studies by the authors and compared with data available in the literature. On the basis of tests carried out for thermally conditioned samples, a combustion mechanism was determined for Zn/KMnO_4_ as a function of temperature. The results show that the combustion process dynamics are independent of equilibrium ratio and limited mainly by diffusion of liquid fuel into the solid oxidising agent. Moreover, it has been revealed that Raman spectroscopy can be effectively used to determine combustion mechanisms for pyrotechnic compositions.

## 1. Introduction

Pyrotechnic delay compositions (PDCs) are chemical systems which are characterised by a combustion velocity that is highly stable and near independent of external conditions. PDCs are used to introduce a specific time interval (“delay”) between two energetic events (e.g., combustion, detonation), with their most common use form being pyrotechnic delay elements found in detonators [1].

A significant number of PDC formulations have been reported in the literature, including recent reports of formulations such as Mn/Bi_2_O_3_ [2], Al/Ni/NiO [3] and even an Al/Si/Bi_2_O_3_, the latter of which is viable as a “pyrotechnic ink” for printing [4]. It should be noted that, despite the favourable properties of these PDCs, they have not found broader application, due to associated issues, such as the relatively high toxicity of Bi_2_O_3_, particularly in the presence of other environmental pollutants [5,6].

Instead, the manufacture of delay detonators relies on “tried and true” PDC formulations that are frequently burdened by similar drawbacks. Currently, two PDC formulations are in wide use: Si/Pb_3_O_4_ and Sb/KMnO_4_ [7,8]. Although the two PDCs offer desirable properties (e.g., high combustion velocities, low amounts of gaseous combustion products), their use poses a significant concern in regard to both human health and the environment.

In order to achieve the required combustion velocity for the Si/Pb_3_O_4_ PDC, the dimensions of the grains of its components need to be on the order of micro- or even nanometers [9,10]. Such fine particles are readily suspended in air, particularly when being transported or poured into mixing units. In the case of silicon, such Si/air suspensions are highly hazardous to human health [11] and constitute an explosive hazard [12]. Pb_3_O_4_, in turn, constitutes a significant threat to human health and a potential source of environmental contamination.

The other PDC, i.e., Sb/KMnO_4_ is also highly problematic, due to the significant toxicity of antimony, as well as its adverse impact on the environment [13,14]. Since potassium permanganate is perceived as a “green” oxidising agent [15], we have focused on replacing antimony with an alternative fuel. In our previous work [16], we have opted to use zinc as such an alternative fuel, due to its minimal (compared to other metallic fuels) toxicity [17] and environmental impact [18], as well as due to its wide availability and modest unit price.

The choice to study the Zn/KMnO_4_ system as a potential PDC came from literature reports claiming that the combustion of this system takes place according to Equation (1) and Equation (2), i.e., without any gaseous combustion products [19].

(1) $$12Zn+10KMnO_{4}\to 2.65K_{2}MnO_{4}+\left(2.35K_{2}O,7.35MnO_{2.05}\right)+12ZnO$$

(2) $$6.8Zn+10K_{2}MnO_{4}\to 5.73K_{3}MnO_{4}+0.5\left(2.9K_{2}O,8.5MnO_{2.1}\right)+6.8ZnO$$

The above chemical reaction equations have been proposed based on the reported mechanism of thermal decomposition of potassium permanganate [20]. It should also be noted that these reaction equations are not adequately balanced, resulting in different amounts of atoms making up the postulated reagents and products (Table A1). This may be due to rounding issues or typographic errors in the original work; however, in the case of Equation (2), the discrepancies exhibit different trends for the atoms, i.e. the products are “deficient” in potassium, while containing an “excess” of manganese and oxygen atoms. Such a difference in trends may indicate that the reaction products may have been mis-identified qualitatively.

No experimental evidence was presented to confirm the veracity of these claims and no literature was cited to support the assumption that zinc present in the system will react with oxygen released through the thermal decomposition of KMnO_4_. These reaction equations imply that combustion of the Zn/KMnO_4_ pyrotechnic composition will yield no gaseous products, which would be extremely desirable from the viewpoint of utilising this composition as a PDC for use in delay detonators.

Our previous investigation has revealed that combustion of Zn/KMnO_4_ in a manometric bomb could produce pressure build-ups of up to 400 kPa [16]. This is direct evidence that Equations (1) and (2) are incorrect, as both the assumption about the combustion of this system yielding no gaseous products and the proposed mechanism of combustion for this system [19] are false.

In light of the above, we have conducted comprehensive investigations of the combustion of the Zn/KMnO_4_ pyrotechnic system. In this work, we present the results of those investigations, proposing an alternative and evidence-based combustion mechanism.

## 2. Results

### 2.1. Raman Spectroscopic Investigations

**Figure 1:**
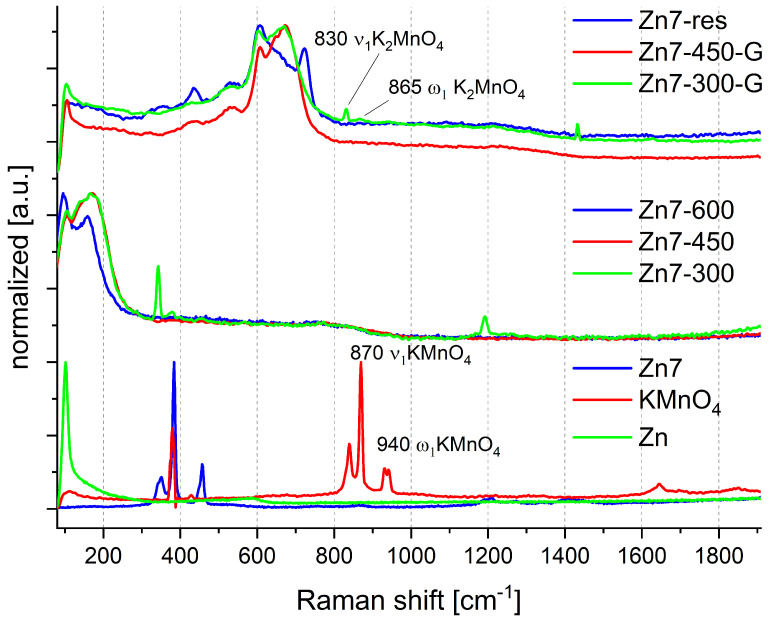
Raman spectra recorded for Zn7 family samples. Signal intensity normalised to the most intense signal. Spectra have been grouped for clarity.

**Figure A1:**
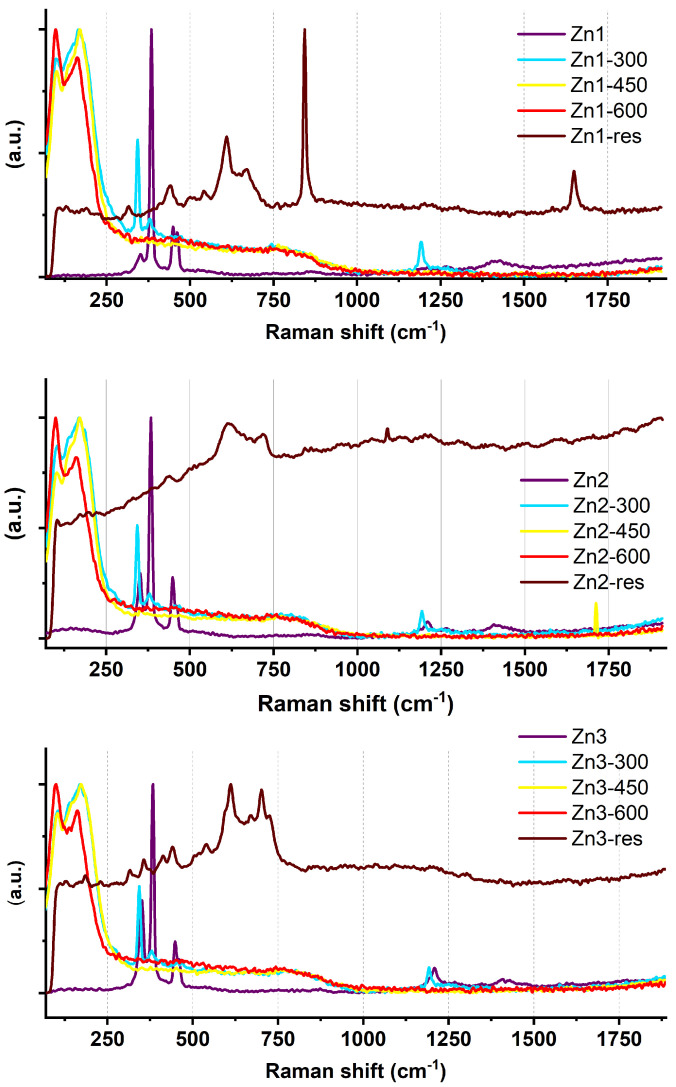
Raman spectra recorded for samples Zn1–Zn3.

**Figure A2:**
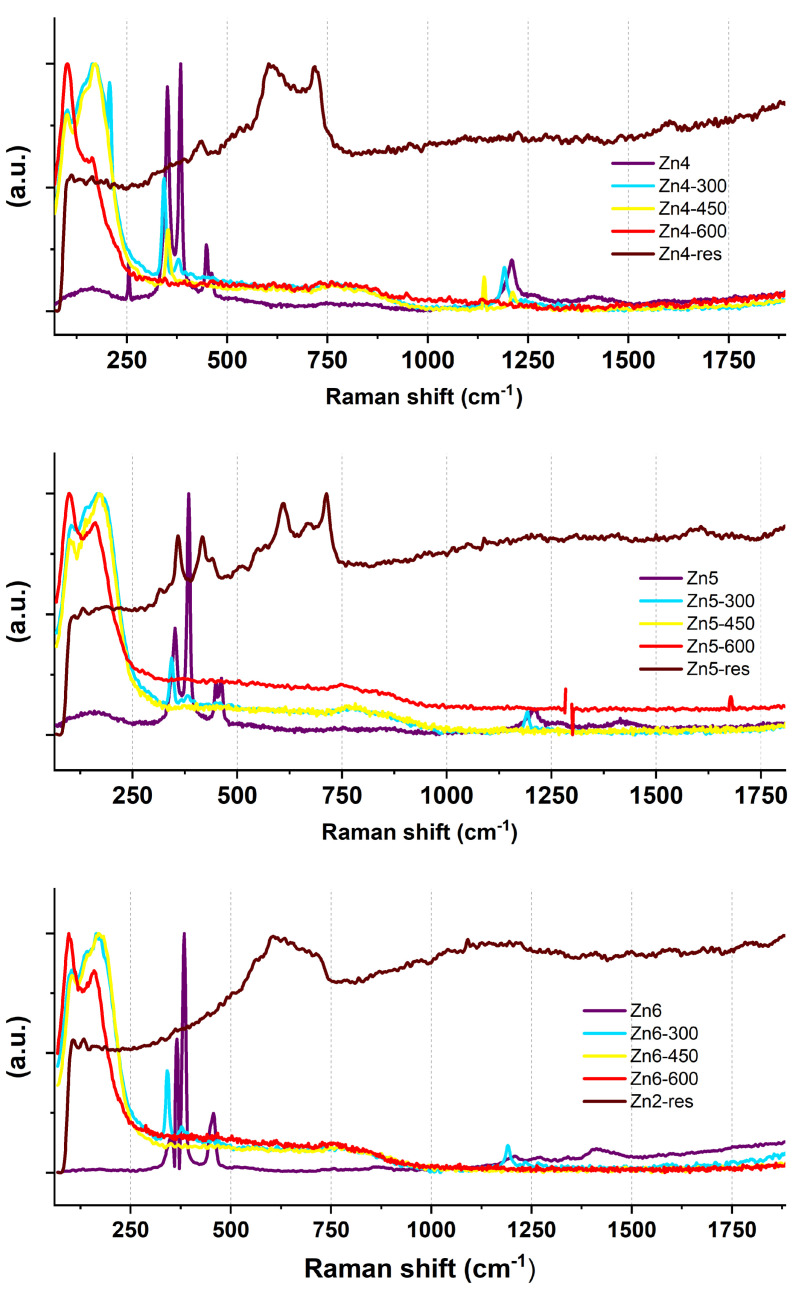
Raman spectra recorded for samples Zn4–Zn6.

The spectra of the PDC components and the Zn7 PDC are summarised in Figure 1, while the Raman spectra of Zn1–Zn6 are shown in Figure A1 and Figure A2.

The site symmetry for KMnO_4_ is C_s_. The crystal has an orthorhombic space group and Z = 4. It would be expected that the Raman spectrum of the solid would exhibit eight lines; one line for ν_1_ 844 cm^−1^, one line for ν_2_ 352 cm^−1^ and three lines each for ν_3_ 905, 913, 918 cm^−1^ and ν_4_ 394, 398, 402 cm^−1^ [21]. It was not possible to record the Raman spectrum of spectrally pure KMnO_4_, because KMnO_4_ undergoes gradual decomposition when exposed to light and air [22], hence resulting in the occurrence of signals originating from the products of this decomposition.

Literature reports zinc oxide as one of the products of combustion of the Zn/KMnO_4_ [19]. Consequently, we have chosen Zn7 for detailed discussion, as it is the sample containing the highest share of Zn from among all the investigated PDC samples. This high share (70 wt.%) of Zn was expected to promote the formation of ZnO rather than mixed metal oxides upon the oxidation of zinc, due to the relatively limited availability of the oxidising agent and its decomposition products.

The occurrence of spontaneous decomposition of KMnO_4_ is seen for all unmodified PDC samples (Zn1–Zn7), as the relevant Raman spectra (Figure 1, Figure A1 and Figure A2) contain signals corresponding to the presence of KMnO_4_ that contains traces of the products of its decomposition products.

In the process of heating the sample, we observe a change in the Raman spectra of the PDC surface. For the samples conditioned at 300 ${}^{{}^{\circ}}$C, there is a partial conversion of manganate(VII) to α-MnO_2_ in which potassium and zinc are intercalated, as evidenced by the response signals at 170 and 210 cm^−1^ [23,24]. It should be noted that only the surface of the grains has such a composition, as either grinding the product or studying the post-combustion residue (Zn7-300-G and Zn7-res, respectively) reveals the presence of other compounds. These compounds include partially decomposed KMnO_4_, likely in the form of manganate(VI), as well as varied manganese oxides: Pyrolusite β-Mn^4+^O_2_ (signal at 755, 660 and 530 cm^−1^), which can transform into cation-doped MnO_2_ (Me^y+^MnO_2_, where Me = K, Zn or Mn) and upon subsequent heating transform into the tetragonal rutile-type β-MnO_2_ band at ∼667 cm^−1^ and α-MnO_2_ type materials with a peak of about 574 and 634 cm^−1^, belonging to A_1g_ spectroscopic signals of a tetragonal hollandite-type framework. In the registered spectrum in the Mn-O range, i.e., 580–740 cm^−1^ [25], there are a number of signals whose shifts indicate the presence of manganese oxides with an oxidation state of 2 to 6. All products produced in the heating process are amorphous, as evidenced by the broadening of the Raman lines.

The presence of K_2_MnO_4_ is evidenced by the signals at 830 cm^−1^ and at 865 cm^−1^ in the Zn7-300-G spectrum and is consistent with literature reports that suggest that K_2_MnO_4_ undergoes decomposition at 540 ${}^{{}^{\circ}}$C in air and at 620 ${}^{{}^{\circ}}$C in nitrogen [20]. The presence of other manganese species, as well as that of Zn may accelerate this decomposition, giving rise to the observed manganese oxides and mixed oxides (intercalation of K, Zn or Mn cations) observed in the spectra of samples conditioned at higher temperatures. The presence of K_2_MnO_4_ only inside the residue particles, in the case of the sample conditioned at 300 ${}^{{}^{\circ}}$C can be explained by the PDC production process. The compositions were crushed inside a circulating crusher. During this process, the coating of one material by the other frequently takes place [26]. The probability of this situation in the Zn/KMnO_4_ system is high, due to the low hardness of zinc [27]. To sum up, the mixing process can result in pressing a thin layer of zinc into the surface of the oxidising agent particles. During subsequent heating, the surface of the oxidising agent particles undergoes decomposition and reaction with the “imprinted” zinc layer, leading to the formation of oxides with lower oxidation states.

### 2.2. X-ray Diffractometry

**Figure 2:**
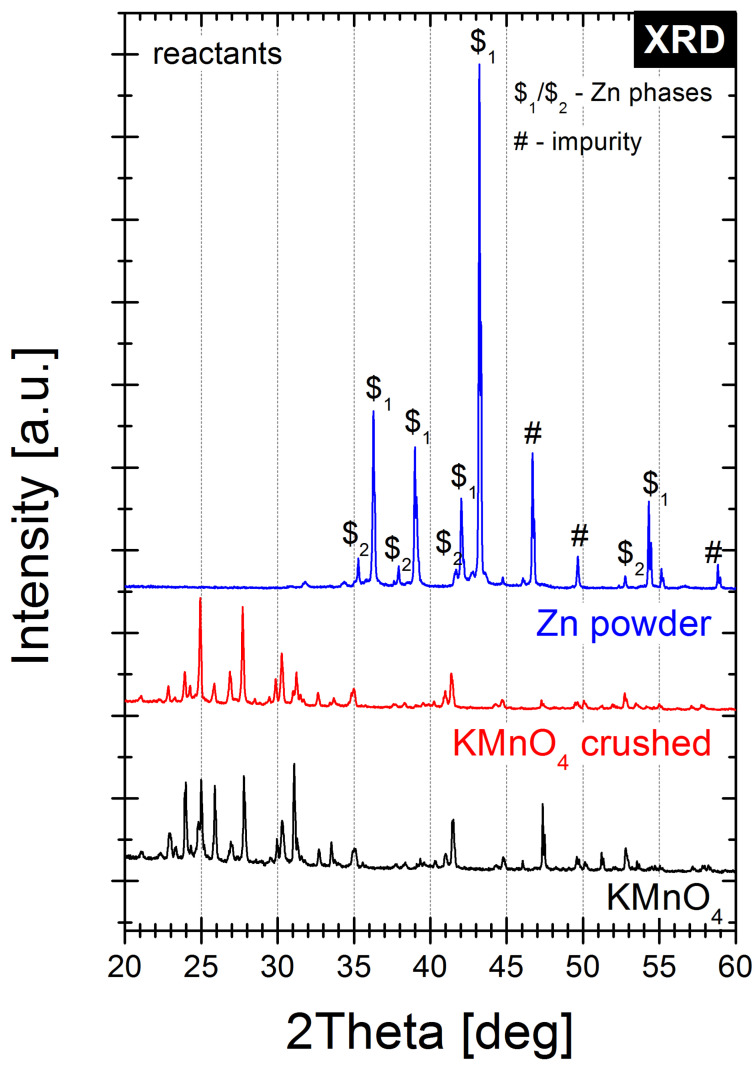
X-ray diffractograms recorded for the reactants used in this work. The main crystalline phases have been marked for clarity.

**Figure 3:**
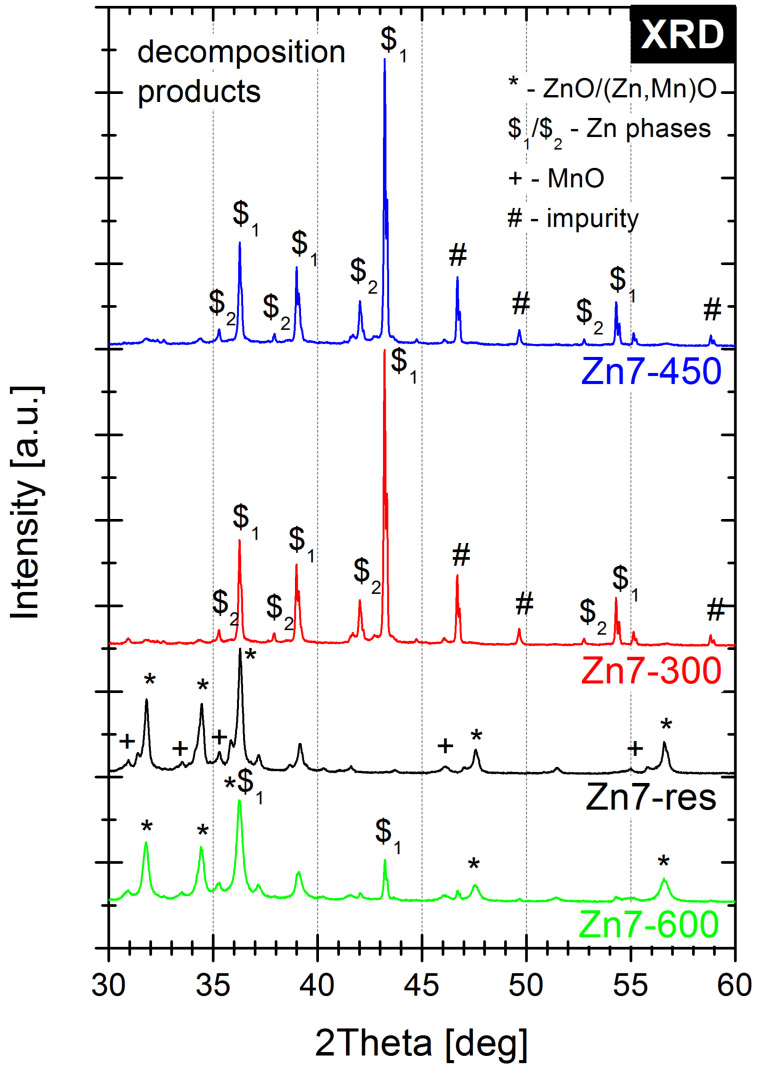
X-ray diffractograms recorded for the Zn7 PDC conditioned at different temperature and the Zn7 post-combustion residue. The main crystalline phases have been marked for clarity.

The XRD patterns of Zn and KMnO_4_ reactants (before and after circulating crushing) and Zn7 family samples decomposition products are illustrated in Figure 2 and Figure 3, respectively, in the 2θ range of 30–60${}^{{}^{\circ}}$ (full range XRD patterns are presented in Figure A4 and Figure A5).

From Figure 2, it is clear that the Zn sample is a multi-component system of two Zn hexagonal phases (PDF Card No. 01-078-9363, PDF Card No. 01-078-7030) of different dimensions, with small amounts of the ZnO phase (PDF Card No. 01-079-5604) evident from the minor Bragg’ peaks and some unidentified peaks defined as “impurity”. The KMnO_4_ powder XRD pattern peaks correspond dominantly to the orthorhombic phase of KMnO_4_ having space group Pnma (Space Group No. 62, PDF Card No. 01-070-3165).

Observations of the PDC decomposition products (Figure 3) suggest that the Zn7-300 and Zn7-450 samples primarily contain unreacted Zn phases with structures described by the same hexagonal phases (PDF Card No. 01-078-9363, PDF Card No. 01-078-7030) as in the utilised Zn powder. In the Zn7-300-G sample, traces of K_3_(MnO_4_)_2_ (PDF Card No. 01-078-3423) were observed, likely arising from interactions between the residual KMnO_4_ and K_2_MnO_4_ produced upon its decomposition. The presence of this compound supports the conclusion drawn from Raman spectroscopic investigations about K_2_MnO_4_ being produced after conditioning at 300 ${}^{{}^{\circ}}$C. Conversely, for Zn7-450-G, this phase is absent. Simultaneously, traces of K_2_(CO_3_)(H_2_O)_1.5_ (PDF Card No. 01-073-0470) are also observed in both Zn7-300-G and Zn7-450-G samples, originating from the contact of the samples with air (containing carbon dioxide and humidity) during their mechanical grinding. Interestingly, for post-combustion residue and Zn7-600 sample, the XRD patterns are identified by the ZnO (PDF Card No. 01-079-0208), (Zn, Mn)O (PDF Card No. 00-062-0414) and MnO (PDF Card No. 01-080-8705) phases. However, for the Zn7-600 sample, the residual unreacted Zn phase remains.

### 2.3. Scanning Electron Microscopy Investigations

**Figure 4:**
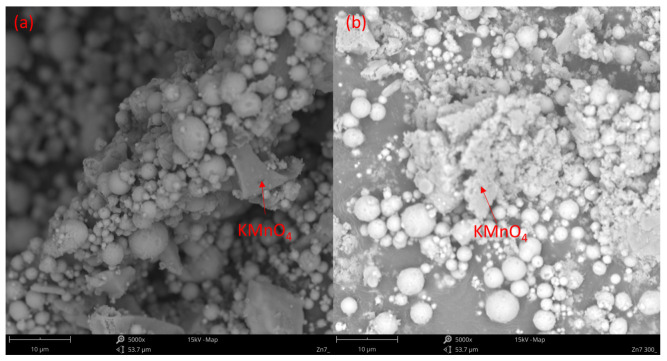
SEM image of (**a**) KMnO_4_ crystals present in the Zn7 sample; (**b**) KMnO_4_ decomposition products present in the Zn7-300 sample. Particles of KMnO_4_ and zinc have been identified based on EDS analysis results and are indicated accordingly.

**Figure 5:**
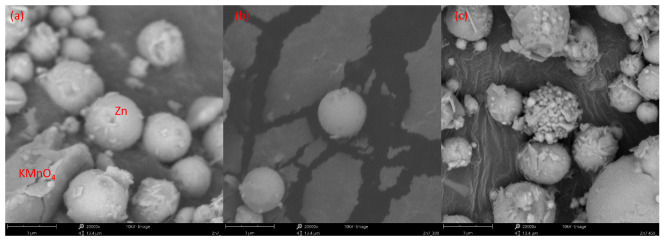
SEM image of Zn particles as a function of thermal conditioning: (**a**) Zn7 sample; (**b**) Zn7-300 sample; (**c**) Zn7-450 sample. Particles of KMnO_4_ and zinc have been identified based on EDS analysis results and are indicated accordingly.

The SEM images recorded for the Zn/KMnO_4_ PDC show that the unconditioned composition is constituted by spherical Zn particles and larger, irregular KMnO_4_ crystallites (Figure 4a and Figure 5a). The images show that the two components are intimately mixed, with no significant degree of phase separation and the dimensions of the Zn particles match the specification of the supplier. Thermal conditioning at 300 ${}^{{}^{\circ}}$C leads to changes in the morphology of the KMnO_4_ crystallites (Figure 4b), which appear to fracture into much finer particles. Conversely, the Zn particles are unaffected at this temperature (Figure 5b). Upon conditioning at 450 ${}^{{}^{\circ}}$C, the Zn particles achieve a composite microstructure, with their surface appearing to be coated by a mixture of smaller particles and irregular crystallites (Figure 5c).

## 3. Materials and Methods

Zinc powder with an average grain size of 3–4 μm (97 wt.% purity), obtained from Selkat S.A (Krakow, Poland), was used as received. Potassium permanganate obtained from LachNer Chemical (Neratovice, Czech Republic) was milled in a circulating crusher for 1 h, followed by sieving to separate grain fractions smaller than 56 μm. The choice of such an oxidant particle size was dictated by the ability to refer of results of the works [19,28], where the authors used an analogous oxidant with a particle size smaller than the 53 μm. Typically, for pyrotechnic systems, the particle size is one of the most important parameters affecting the combustion process [29].

### 3.1. Preparation of PDC Samples

The samples of the Zn/KMnO_4_ were prepared exactly as in our previous work [16], that is, the components were dried, weighed in the relevant amounts and mixed by brushing the PDC through a sieve, followed by drying the PDC. Samples with a zinc content ranging from 35 wt.% to 70 wt.% (Table 1) were prepared.

#### 3.1.1. Thermal Conditioning of Samples

**Figure A3:**
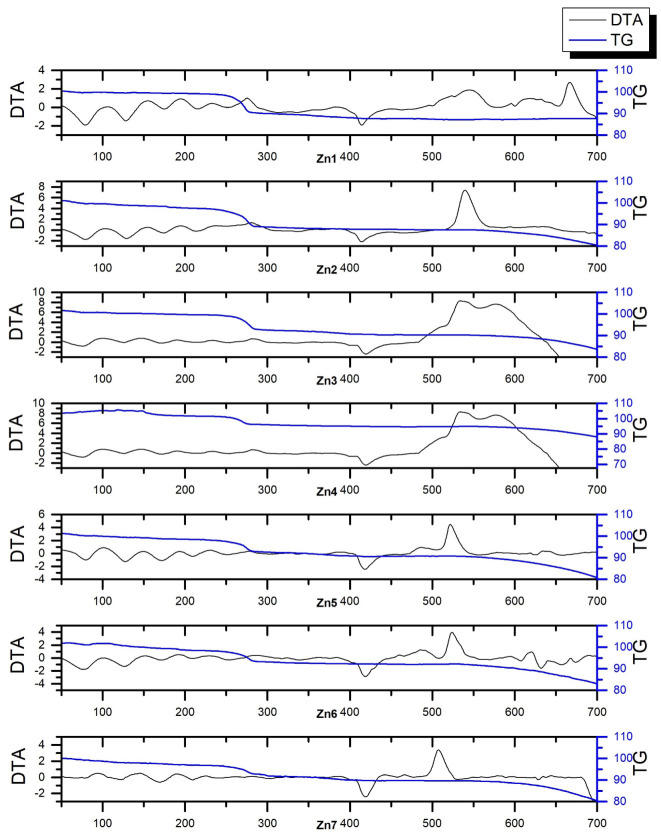
DTA and TG curves for Zn1–Zn7 samples. Reprinted from [16] under a Creative Commons CC-BY license.

**Figure A4:**
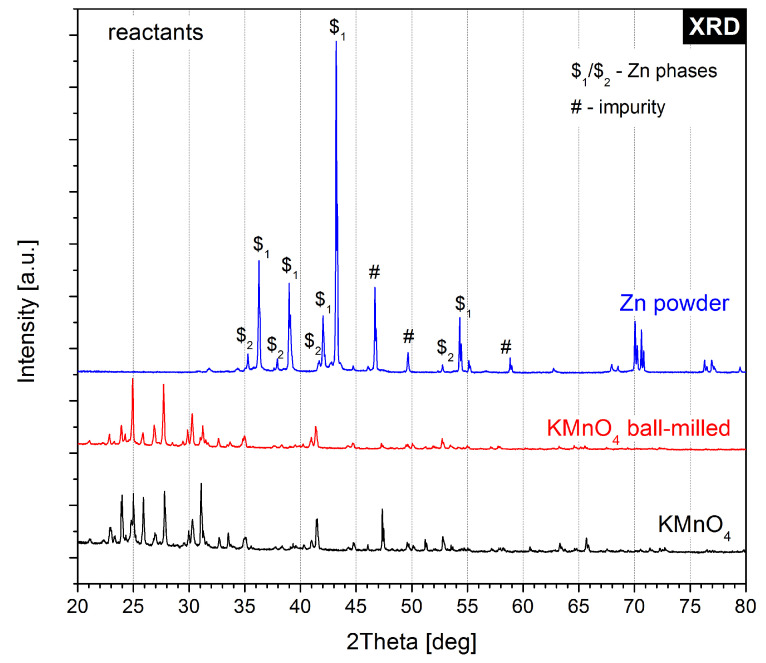
Full-range X-ray diffractograms recorded for the reactants used in this work. The main crystalline phases have been marked for clarity.

**Figure A5:**
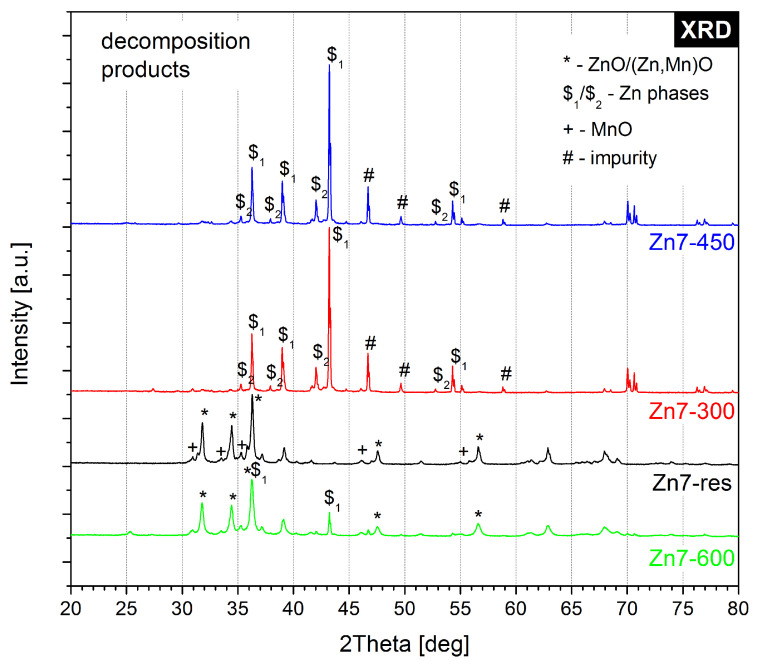
Full-range X-ray diffractograms recorded for the Zn7 PDC conditioned at different temperature and the Zn7 post-combustion residue. The main crystalline phases have been marked for clarity.

Samples from Zn1 to Zn7 were conditioned thermally in an oven, under argon atmosphere for 24 h, using a heating rate of 20 K/min for all samples. The cooling rate was equal to 0.5 K/min. Based on the results of thermogravimetric measurements (Figure A3) reported in our previous work [16], three conditioning temperatures were selected:

- 300 ${}^{{}^{\circ}}$C—the samples all showed an initial mass loss step at approx. 270 ${}^{{}^{\circ}}$C, which we attributed to the decomposition of the permanganate anion. Hence, this temperature was chosen to identify the products of the reaction underlying this mass loss step.
- 450 ${}^{{}^{\circ}}$C—zinc is known to have a melting point of 419.5 ${}^{{}^{\circ}}$C [30]. This temperature point was chosen to allow for the investigation of whether the fusion of zinc and, therefore, significantly increased surface of contact between the reagents would lead to the occurrence of any reactions in the condensed (solid/liquid) phase.
- 600 ${}^{{}^{\circ}}$C—the samples typically underwent ignition at approx. 500 ${}^{{}^{\circ}}$C, with the exact ignition temperature being dependent on the ratio of the two reagents in the sample. This temperature point was selected as a “blank sample” for comparison with the samples of post-combustion residues, for whom oxidation by atmospheric oxygen is also a factor that alters their chemical composition.

Samples subjected to thermal conditioning are referred to by their composition label (e.g., Zn1) appended by the conditioning temperature, i.e., Zn1-300 refers to a PDC containing 35 wt.% zinc that was conditioned at 300 ${}^{{}^{\circ}}$C.

In order to verify whether the surface of the particles of the thermally conditioned samples (analysed by Raman spectroscopy) had the same composition as their interior, we have subjected parts of the Zn7-300 and Zn7-450 samples to 5 min of mechanical grinding. These samples have been appended with “G” to indicate this fact, e.g., Zn7-300-G.

#### 3.1.2. Post-Combustion Residues

Post-combustion residues were produced by combustion of PDC formulations in combustion cell with constant volume of 45 cm^3^. The detailed testing process has been explained in our previous work [16]. Typically, a cylindrical aluminium shell equipped with nichrome resistant (0.95–1 Ω) wire was filled with a 400 mg charge of composition and placed in the combustion cell. Each PDC formulation was ignited using the hot-wire method by applying a DC pulse of 4.25 A. The samples of residues are named by their respective composition label (e.g., Zn1) appended by “res”, i.e., Zn7-res refers to the residue obtained from the combustion of a PDC containing 70 wt.% zinc.

### 3.2. Scanning Electron Microscopy

The morphology and chemical composition of the post-combustion residues were investigated using a Phenom ProX (Waltham, MA, USA) equipped with scanning electron microscope (SEM), and an X-ray energy dispersive spectrometer. The basic SEM operation parameters were that the working distance was 10 mm, the acceleration voltages of the incident electron were 5 kV and 15 kV, the electronic beam spot size was 5 and the current intensity of the incident electronic beam was about 95 μA.

### 3.3. Raman Spectroscopy

Raman spectroscopy was performed using a Raman microscope (inVia Renishaw, Wotton-under-Edge, UK), which was equipped with a CCD detector and using red (633 nm) laser excitation. Spectra were recorded in a fixed range of 100–1900 cm^−1^. All measurements were made in a backscattering geometry using a 50× microscope objective with a numerical aperture value of 0.75, providing scattering areas of 1 μm^2^. Single-point spectra were recorded with a laser power of 1.7 mW, with 4 cm^−1^ resolution and 10 s acquisition times with 10 accumulations. The limited power and acquisition time were employed so as to minimise the decomposition of KMnO_4_ caused by irradiation [22].

### 3.4. X-ray Diffractometry

X-ray diffraction (XRD) with Cu Kα radiation (λ = 1.54183 Å) was carried out to identify the crystalline phases using Rigaku MiniFlex 600 (Rigaku Co., Tokyo, Japan) at room temperature, using a one-dimensional detector (Rigaku D/teX Ultra 250). The X-ray tube was operated at 40 kV and 15 mA. Additional measurements parameters are 2θ range 3–80${}^{{}^{\circ}}$, IHS slit = 10 mm, Soller slits = 2.5${}^{{}^{\circ}}$, DS slit = 1.25${}^{{}^{\circ}}$, scanning step size 0.01${}^{{}^{\circ}}$, and exposure time at each point of 1.67 s without sample rotation.

## 4. Discussion

Based on the obtained results, we can conclude that the first reaction step is the decomposition of potassium permanganate with the release of gaseous oxygen. The released oxygen, contrary to literature reports [19], does not react with zinc and does not produce zinc oxide (ZnO). This is supported by the fact that the Raman spectra of the Zn1-300–Zn7-300 samples contain no signals that can be attributed to the presence of ZnO, even in the case of the Zn7-300-G sample. Instead, the decomposition of KMnO_4_ is supported by Raman signals attributed to cation-doped MnO_2_ in the Mn7-300 and to MnO_4_^2−^ in the Mn7-300-G samples. This is also in line with the observed changes in the morphology of the samples, i.e., the fragmentation of KMnO_4_ particles into finer grains, seen upon comparison of the SEM images for Zn7 (Figure 4a) and Zn7-300 (Figure 4b).

The next step of the combustion process is the fusion of zinc at 419.5 ${}^{{}^{\circ}}$C. This significantly increases the contact surface between the reagents and leads to the stratification of the system, due to differences in the densities (Table 2) of its constituents. This is evidenced by the significant difference between the composition (as found by Raman spectroscopy) of the surface and interior of the PDC particles conditioned at 450 ${}^{{}^{\circ}}$C (Zn7-450 vs. Zn7-450-G). The stratification of the PDC is also supported by the fact that the zinc grains observed by SEM for Zn7-300 gain a “Raffaello-like” microstructure in Zn7-450 (Figure 5c). Furthermore, the XRD study showed that Zn7-300 and Zn7-450 samples contain mainly unreacted fuel, without the presence of ZnO or mixed oxides, which is in line with the results of Raman spectroscopic investigations.

Apart from the formation of the stratified reaction system, conditioning the samples at 450 ${}^{{}^{\circ}}$C primarily leads to the decomposition of manganates(VI). Interestingly, where the surface of the particles constituting the samples typically contains cation-doped MnO_2_, their interior appears to contain a mixture of somewhat amorphous manganese oxides (β-MnO_2_, Mn_2_O_3_ and Mn_3_O_4_). It should be noted that no signals that could be attributed to zinc oxide were observed by Raman spectroscopy at this point. Consequently, it is unlikely for any significant amounts of zinc to have been oxidised after conditioning at 450 ${}^{{}^{\circ}}$C. Even if oxidation of zinc had been taking place, it would proceed via reaction of zinc with the manganates (observed at 300 ${}^{{}^{\circ}}$C), resulting in mixed zinc-manganese oxides, such as the observed cation-doped manganese(IV) oxide.

The third stage of combustion, taking place at approx. 500 ${}^{{}^{\circ}}$C, is associated with the intensification of reactions between zinc and the manganese oxides, resulting in a significant exothermic effect, as evidenced via thermogravimetric results (Figure A3) presented in our previous work [16]. These reactions likely take place between the cation-doped MnO_2_ and zinc, as seen by the observed decrease in the intensity of the cation-doped MnO_2_ Raman signals, as well as by the lack of any signals that can be attributed to ZnO, which would be expected if zinc were to be oxidised by oxygen originating from the decomposition of manganates or manganese oxides. XRD results for the Zn7-600 sample confirm the aforementioned conclusions. It should be noted that the XRD signals occurring from zinc oxide, manganese oxide and their mixed oxides largely coincide and may originate from the mixed oxides identified via Raman spectroscopy. It was considered whether the ZnO phase may be present within combustion products agglomerates and not visible in Raman experiments due to the low penetration of visible light through the samples. Raman study of the samples subjected to grinding (Zn7-300-G and Zn-450-G), which would be expected to reveal the insides of these agglomerates, did not reveal the presence of ZnO, making the existence of its phase in any appreciable amounts unlikely.

The above proposed mechanism matches the composition of the post-combustion residues of the PDC to a significant extent. The sole deviation, i.e., the presence of unreacted manganates in the residues, stems from the fact that combustion is orders of magnitude more rapid than our thermal conditioning procedure, giving rise to an inhomogeneous distribution of temperatures across the bulk of the composition. This in turn may result in areas in which the exposure to high temperatures is insufficient (in terms of both duration and attained temperature) to achieve complete decomposition of manganates to manganese oxides. This effect could be related also to macroscopic level inhomogeneities that may be introduced upon processing the PDC in bulk.

In light of the above, the following sequence of reactions and physical processes is proposed to underlie the combustion of the Zn/KMnO_4_ PDC. The reactions have not been balanced, as the focus of this study is to provide a qualitative rather than quantitative description of this PDC system:

At or below 300 ${}^{{}^{\circ}}$C:

(3) $$KMnO_{4}\to K_{2}MnO_{4}+Me_{x}^{y+}MnO_{2}+MnO_{2}+Mn_{2}O_{3}+Mn_{3}O_{4}+O_{2}↑$$

At or below 450 ${}^{{}^{\circ}}$C:

(4) $$Zn_{\left(s\right)}\to Zn_{\left(l\right)}$$

(5) $$K_{2}MnO_{4}\to MnO_{2}+Mn_{2}O_{3}+Mn_{3}O_{4}+K_{x}MnO_{2}$$

At or above 450 ${}^{{}^{\circ}}$C:

(6) $$Zn_{\left(l\right)}+MnO_{z}\to ZnO\cdot MnO_{z^{\prime }}$$

where:

Me^y+^ = K^+^, Zn^2+^ or Mn^2+^;

MnO_*z*_ = any of the manganese oxides, for which *z* > 1;

MnO*z*${}^{\prime }$ = relevant reduced form of the manganese oxide taking part in the reaction. 

In terms of a macroscopic scale description of the combustion mechanism of this PDC, the following hypothesis is proposed. The initial decomposition of KMnO_4_ (at approximately 270 ${}^{{}^{\circ}}$C) leads to the release of significant amounts of oxygen from the system. Depending on the size of the charge, cover material and heating rate, the released oxygen can take part in combustion reactions; for example, at the combustion front of PDC elements, typically heating rates are in order of hundreds of K/min [33]. After this process, the remaining oxidising agent is characterised by high porosity.

Zinc melting, occurring at 419.5 ${}^{{}^{\circ}}$C, results in the formation of a system in which the continuous phase of zinc contains solid oxidising agent inclusions. This causes the pre-ignition process to take place only on the surface of the oxidising agent particles, in conditions of a significant excess of fuel. The layer formed during that process partially limits further contact between the fuel and the oxidising agent particles. Most likely, this reaction already takes place at temperatures around 450 ${}^{{}^{\circ}}$C. Diffusion of fuel deep into the pores of the oxidising agent particles most probably occurs on a longer time scale and is strongly limited by mass transport efficiency. The fuel/oxidising agent ratio inside the pores differs significantly from the earlier reaction at the surface of the oxidising agent particles. This would explain the difference in product composition between the surface and interior of the particles of the investigated samples.

Differences between XRD and Raman spectroscopic investigations are related to the penetration depth of the two utilised types of electromagnetic radiation, with much higher penetration taking place in the case of XRD. Although the limited sample penetration in Raman spectroscopy is a significant issue for such analyses, it can be ameliorated to an extent by grinding the samples. Intercalation of ZnO with cations (primarily Mn cations) does not need to interfere with the crystalline lattice in comparison with non-intercalated ZnO, resulting in XRD analyses being inconclusive as to whether ZnO or mixed oxides were formed, particularly when signals attributed to manganese oxides also being observed. Although this question can be resolved through Raman spectroscopy, particular attention should be dedicated to the possible presence of any core–shell structures, whose interior may not be observable by Raman spectroscopy. In the case of the investigated samples, the presence of ZnO within the interior of the particle agglomerates constituting the samples was found to be unlikely, based on comparison with the ground samples, but cannot be entirely excluded.

The abovementioned hypothesis points to an interesting conclusion. The composition of the combustion products is quite similar, regardless of fuel content, even though the tested compositions represented a wide spread of fuel contents. It allows us to propose the existence of constant reaction kinetics, limited primarily by the capacity of liquid zinc to permeate into the oxidising agent particles.

Discrepancies in combustion velocity or pressure parameters, proved in our previous work [16], are mainly related to the content of zinc not involved in the actual combustion reaction. During closed vessel combustion tests, the released oxygen could be partially connected with pressure rise, but most likely the thermal decomposition rate of KMnO_4_ is not fast enough in comparison with real heating rates on combustion front. The main pressure peak is related to the combustion reactions and excess fuel vapourisation, which is highly visible for systems with strongly negative oxygen balance values. The pressure tests, described in our previous work [16] were performed in air atmosphere, inside a large volume vessel, whereas mass exchange conditions inside a delay element are completely different. An increase in zinc content up to a certain value, leads to an increase in combustion velocity and pressure parameters, due to improved heat transfer conditions and presence of zinc vapours in reaction products. Above this limit, the amount of heat required to melt and vaporise zinc outweighs this beneficial effect, leading to a decrease in previously mentioned parameters.

## 5. Conclusions

To summarise, in this work, we have presented evidence for the incongruity of the combustion mechanism of the Zn/KMnO_4_ PDC proposed in the literature with experimental observations. We have proposed an alternate initial mechanism, based on our ability to identify the products of reactions taking place up to each of the selected temperatures. This mechanism both accounts for the results of experimental testing reported in our previous work (thermogravimetry, pressurisation rate upon combustion in a closed vessel, combustion velocity) of the composition and is congruent with the observed composition of the products of combustion of this PDC in open air. This mechanism also accounts for the fact that no particular stoichiometric ratio of fuel to the oxidising agent, yielding a PDC with noticeably higher combustion parameters, appears to exist for this system, due to its combustion being diffusion-limited, as discussed above.

Manganese forms a broad variety of substances, in the form of various manganates, oxides, compounds with other transition metals and oxides doped with cations of other metals. Unfortunately, the number of literature reports dedicated to the identification of and differentiation between these compounds is very limited. Due to this, in some cases, we have been unable to pinpoint the exact reaction products and were forced to limit our insight to a group of such compounds. Formulating a full qualitative and quantitative description of the combustion mechanism of the Zn/KMnO_4_ PDC is highly relevant to both the design of PDCs in general and to the development of this PDC in particular. Such a mechanical description, however, is nigh-impossible based on the current state of the art, and an in-depth exploration of the relevant compounds of manganese is called for.

## Figures and Tables

**Table 1:** Composition and sample naming scheme of the investigated Zn/KMnO_4_ PDC formulations.

Composition Label	Zn1	Zn2	Zn3	Zn4	Zn5	Zn6	Zn7
**Zn [wt.%]**	35	45	50	55	60	65	70
**KMnO_4_ [wt.%]**	65	55	50	45	40	35	30

**Table 2:** Densities of the components and possible products of combustion of the Zn/KMnO_4_ PDC.

Element or Compound	Density [g·cm^−3^]	Ref.
Zn	7.134	[31]
ZnO	5.6	
MnO	5.37	
Mn_3_O_4_	4.84	
Mn_2_O_3_	5	
MnO_2_	5.08	
KMnO_4_	2.7	
K_2_MnO_4_	2.81	[32]

## Data Availability

The data presented in this study are available on request from the authors.

## Appendix A

**Table A1:** Atom balance in Zn/KMnO_4_ PDC combustion equations proposed in literature [19].

Equation (1)		
**Atom**	**Reagents**	**Products**
Zn	12	12
K	10	10
Mn	10	10
O	40	40.0175
**Equation (2)**		
**Atom**	**Reagents**	**Products**
Zn	6.8	6.8
K	20	20.09
Mn	10	9.98
O	40	40.095

## References

[1] Shaw A., Poret J., Groven L., Koenig J., Brusnahan J. (2023). Pyrotechnic Delay Element Device. US Patent.

[2] Tichapondwa S.M., Guo S., Roux W.E. (2021). Performance of Mn/Bi_2_O_3_ pyrotechnic time delay compositions. Cent. Eur. J. Energetic Mater..

[3] Guo S., Focke W.W., Tichapondwa S.M. (2020). Al-Ni-NiO Pyrotechnic Time-Delays. Propellants Explos. Pyrotech..

[4] Bell T.M., Williamson D.M., Walley S.M., Morgan C.G., Kelly C.L., Batchelor L. (2020). An Assessment of Printing Methods for Producing Two-Dimensional Lead-Free Functional Pyrotechnic Delay-Lines for Mining Applications. Propellants Explos. Pyrotech..

[5] Akhtar M.J., Ahamed M., Alhadlaq H. (2023). Bismuth Oxide (Bi_2_O_3_) Nanoparticles Cause Selective Toxicity in a Human Endothelial (HUVE) Cell Line Compared to Epithelial Cells. Toxics.

[6] Ahamed M., Akhtar M.J., Khan M.A.M., Alhadlaq H.A. (2021). Co-exposure of Bi_2_O_3_ nanoparticles and bezo [a] pyrene-enhanced in vitro cytotoxicity of mouse spermatogonia cells. Environ. Sci. Pollut. Res..

[7] Ren H., Jiao Q., Chen S. (2010). Mixing Si and carbon nanotubes by a method of ball-milling and its application to pyrotechnic delay composition. J. Phys. Chem. Solids.

[8] Beck M.W., Brown M.E. (1986). Modification of the burning rate of antimony/potassium permanganate pyrotechnic delay compositions. Combust. Flame.

[9] Zhang B., Huang C., Yan S., Li Y., Cheng Y. (2013). Enhanced reactivity of boron, through adding nano-aluminum and wet ball milling. Appl. Surf. Sci..

[10] Gabdrashova S., Tulepov M., Korchagin M., Sassykova L., Abdrakova F.Y., Bexultan Z.B., Aitenov Y., Toktagul S., Baiseitov D. (2021). Development of pyrotechnic delay mixtures based on a composite material hardened with carbon nanotubes. Dig. J. Nanomater. Biostruct.

[11] Leonova M., Timofeeva S., Murzin M. Dust load in silicon production and occupational risks. Proceedings of the IOP Conference Series: Materials Science and Engineering.

[12] Skjold T. An experimental investigation of flame propagation in clouds of silicon dust dispersed in air, hydrogen-air mixtures, and hybrid Si-H2-air mixtures. Proceedings of the Twenty-Fifth International Colloquium on the Dynamics of Explosions and Reactive Systems.

[13] Boreiko C.J., Rossman T.G. (2020). Antimony and its compounds: Health impacts related to pulmonary toxicity, cancer, and genotoxicity. Toxicol. Appl. Pharmacol..

[14] Zhang P., Wu T.L., Ata-Ul-Karim S.T., Ge Y.Y., Cui X., Zhou D.M., Wang Y.J. (2020). Influence of soil properties and aging on antimony toxicity for barley root elongation. Bull. Environ. Contam. Toxicol..

[15] Singh N., Lee D.G. (2001). Permanganate: A green and versatile industrial oxidant. Org. Process Res. Dev..

[16] Polis M., Szydło K., Jarosz T., Procek M., Skóra P., Stolarczyk A. (2022). Investigation of combustion of KMnO_4_/Zn pyrotechnic delay composition. Materials.

[17] Agnew U.M., Slesinger T.L. (2020). Zinc Toxicity.

[18] McGrath S.P., Chaudri A.M., Giller K.E. (1995). Long-term effects of metals in sewage sludge on soils, microorganisms and plants. J. Ind. Microbiol..

[19] Tribelhorn M.J., Venables D.S., Brown M.E. (1995). Combustion of some zinc-fuelled binary pyrotechnic systems. Thermochim. Acta.

[20] Herbstein F., Ron G., Weissman A. (1971). The thermal decomposition of potassium permanganate and related substances. Part I. Chemical aspects. J. Chem. Soc. A Inorg. Phys. Theor..

[21] Clark R.J., Dines T.J., Doherty J.M. (1985). Resonance Raman spectroscopy of the manganate (VI) ion: Band excitation profiles and excited-state geometry. Inorg. Chem..

[22] Hu X., Shi L., Zhang D., Zhao X., Huang L. (2016). Accelerating the decomposition of KMnO_4_ by photolysis and auto-catalysis: A green approach to synthesize a layered birnessite-type MnO_2_ assembled hierarchical nanostructure. RSC Adv..

[23] Gao T., Fjellvåg H., Norby P. (2009). A comparison study on Raman scattering properties of *α*-and *β*-MnO_2_. Anal. Chim. Acta.

[24] Julien C., Massot M. (2003). Raman spectroscopic studies of lithium manganates with spinel structure. J. Phys. Condens. Matter.

[25] Bernardini S., Bellatreccia F., Casanova Municchia A., Della Ventura G., Sodo A. (2019). Raman spectra of natural manganese oxides. J. Raman Spectrosc..

[26] Zygmunt B., Wilk Z. (2008). Formation of jets by shaped charges with metal powder liners. Propellants Explos. Pyrotech. Int. J. Deal. Sci. Technol. Asp. Energetic Mater..

[27] Liberati A.C., Che H., Vo P., Yue S. (2021). Influence of secondary component hardness when cold spraying mixed metal powders on carbon fiber reinforced polymers. J. Therm. Spray Technol..

[28] Tribelhorn M.J., Venables D.S., Brown M.E. (1995). A thermoanalytical study of some zinc-fuelled binary pyrotechnic systems. Thermochim. Acta.

[29] Focke W.W., Tichapondwa S.M., Montgomery Y.C., Grobler J.M., Kalombo M.L. (2019). Review of gasless pyrotechnic time delays. Propellants Explos. Pyrotech..

[30] Chase M.W., NISO (1998). NIST-JANAF Thermochemical Tables.

[31] Haynes W.M. (2016). CRC Handbook of Chemistry and Physics.

[32] Herbstein F. (1960). Crystallographic data for potassium manganate K_2_MnO_4_. Acta Crystallogr..

[33] Miklaszewski E.J., Shaw A.P., Poret J.C., Son S.F., Groven L.J. (2014). Performance and aging of Mn/MnO_2_ as an environmentally friendly energetic time delay composition. ACS Sustain. Chem. Eng..

